# Overexpression of CHD1L is associated with poor survival and aggressive tumor biology in esophageal carcinoma

**DOI:** 10.18632/oncotarget.18830

**Published:** 2017-06-29

**Authors:** Ze-Han Liu, Qi Zhang, Yi-Jie Ding, Ying-Hui Ren, Hui-Peng Yang, Qing Xi, Ying-Nan Cheng, Guo-Lin Miao, Hong-Kun Liu, Cai-Xia Li, Wen-Qiang Yan, Yan Li, Zhenyi Xue, Lijuan Zhang, Xin-Ye Li, Chen-Long Zhao, Yurong Da, Xian-Zhong Wu, Jun-Qiang Chen, Rongxin Zhang, Zhi-Gang Li

**Affiliations:** ^1^ Nankai Clinical College, Tianjin Medical University, Tianjin, P.R. China; ^2^ Institute of Integrative Medicine Therapy for Acute Abdominal Diseases of Tianjin, Nankai Hospital, Tianjin, P. R. China; ^3^ Laboratory of Immunology and Inflammation, Department of Immunology, Key Laboratory of Immune Microenvironment and Diseases of Educational Ministry of China, Basic Medical College, Tianjin Medical University, Tianjin, P.R. China; ^4^ First Central Clinical College, Tianjin Medical University, Tianjin, P.R. China; ^5^ Department of Thoracic Surgery, Nankai Hospital, Nankai District, Tianjin, P. R. China; ^6^ General Hospital, Tianjin Medical University, Tianjin, P.R. China; ^7^ Department of Radiotherapy, Fujian Provincial Tumor Hospital, Affiliated Tumor Hospital of Fujian Medical University, Fuzhou, P.R. China; ^8^ Laboratory of Immunology and Inflammation, Guangdong Pharmaceutical University, Guangzhou, P.R. China; ^9^ Hainan Cancer Hospital, Affiliated Cancer Hospital of Hainan Medical College, Haikou City, P.R. China

**Keywords:** esophageal carcinoma, CHD1L protein, apoptosis, migration, prognosis

## Abstract

Esophageal carcinoma (EC) is a malignancy with high metastatic potential. Chromosomal helicase/ATPase DNA binding protein 1-like (CHD1L) gene is a newly identified oncogene located at Chr1q21, and it is amplified in many solid tumors. However, the status of CHD1L protein expression in EC and its clinical significance is uncertain. This study was designed to investigate the significance of CHD1L expression in human EC and its biological function in EC cells. The expression of CHD1L was examined by immunohistochemistry in 191 surgically resected ECs. The associations between CHD1L expression and clinical pathological parameters and the prognostic value of CHD1L were analyzed. Western blot analysis showed that CHD1L was overexpressed in EC cell lines. In addition, positive CHD1L expression was strongly related to advanced clinical stage (*P*<0.01), and lymph node metastasis (*P*<0.01) of EC. The Kaplan-Meier curve indicated that high expression of CHD1L may result in poor prognosis of EC patients (*P*<0.01), and multivariate analysis showed that CHD1L overexpression was an independent predictor of overall survival. Furthermore, suppression of CHD1L in EC cells increased apoptosis and decreased cell proliferation invasion ability. Our results suggest that CHD1L is a target oncogene with the potential to serve as a novel prognostic biomarker in EC pathogenesis.

## INTRODUCTION

Esophageal cancer (EC) is one of the most frequent malignant tumors and is the sixth most common cause of death worldwide [1, 2]. Like other solid tumors, the development of EC often involves the acquisition of genetic alterations [3–5] and corresponding changes in protein expression that modify normal growth control and survival pathways [6, 7]. Thus, exploring potential novel biomarkers of EC will help to establish the primary diagnosis and treatment regimens, as well as determine the prognosis for EC patients. The chromodomain helicase/ATPase DNA binding protein 1-like gene (CHD1L), also known as amplified in liver cancer 1 gene (ALC1), was recently identified as a target oncogene within the 1q21 amplicon in hepatocellular carcinoma (HCC) [8–10]. In addition, CHD1L has been reported as a novel prognostic biomarker in several types of solid tumors, including prostate cancer, lung cancer, breast cancer, gastric cancer, colorectal cancer, bladder cancer, nasopharyngeal carcinoma, and ovarian cancer [11–17]. However, the expression of CHD1L and its significance in EC has not been well documented and remains uncertain. In this study, we examined the expression patterns of CHD1L in EC tissues and analyzed the relationship between CHD1L expression and the clinicopathological factors of EC. Furthermore, we investigated the possible role of CHD1L on cell proliferation, apoptosis, and migration in EC. Our data suggest that CHD1L overexpression is likely associated with aggressive tumor biology in EC. CHD1L might be a novel biomarker and therapeutic target for EC patients, and its clinicopathological and biology significance was evaluated.

## RESULTS

### CHD1L is overexpressed in EC tissues

**Figure 1:**
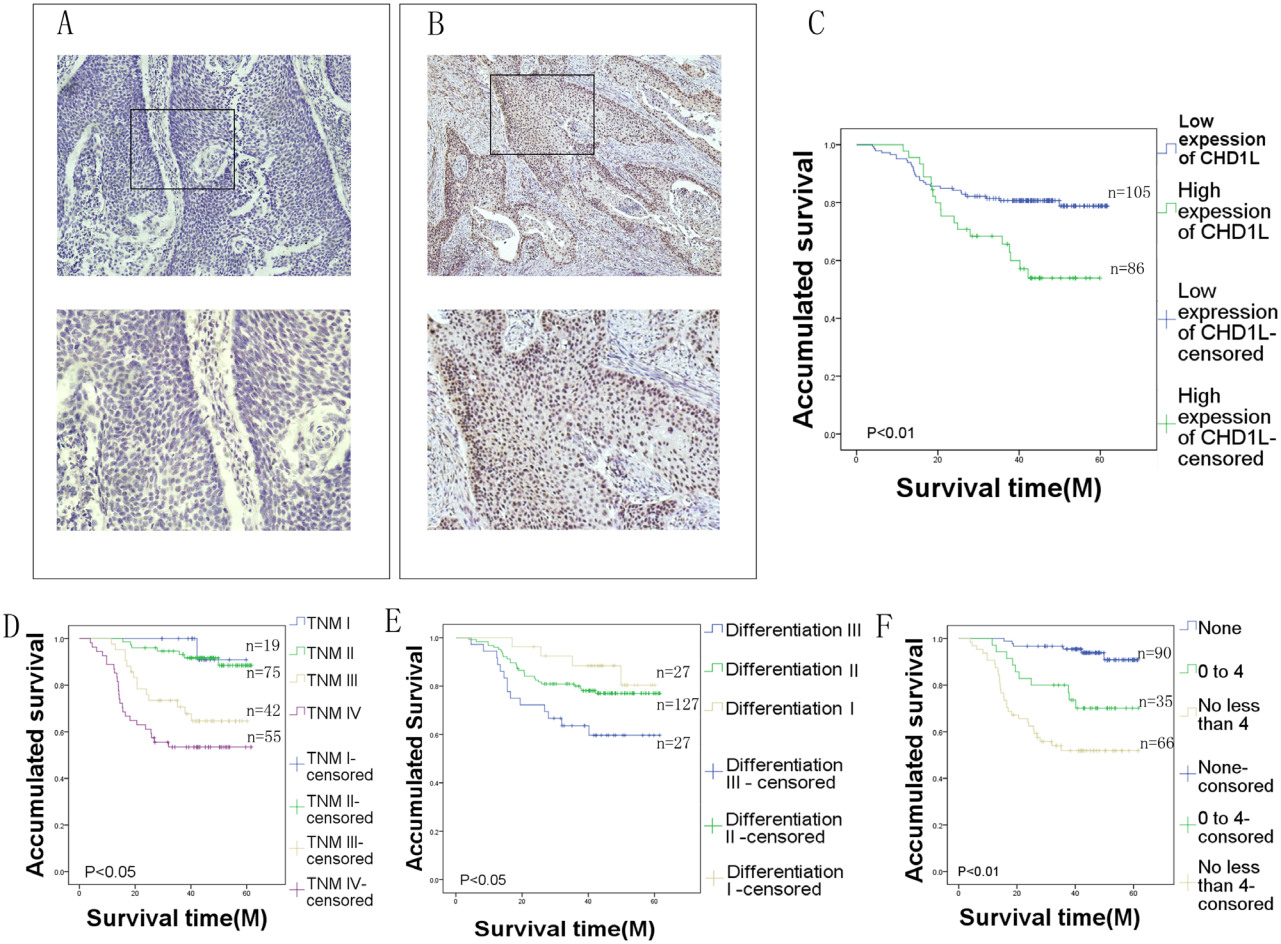
Immunohistochemical analysis of CHD1L in EC tissues **(A)** Low CHD1L expression in EC tissues (105 cases). **(B)** High CHD1L expression in EC tissues (86 cases) (upper panel, ×40; lower panel, ×200). **(C)** Survival curve for 191 EC patients according to CHD1L protein expression status (log-rank test). Overall survival of all patients with EC: low expression, n=105 cases, mean time=40.6 months; high expression, n=86, mean time=35.2 months. **(D, E,** and **F)** Survival curve for 191 EC patients according to clinical stage, differentiation, and lymph node metastasis.

For CHD1L protein IHC staining in EC tissues, positive staining was seen primarily in the nuclei within tumor cells, though occasionally yellowish-brown granules were also observed in the cytoplasm (Figure 1A&1B). Only the intensity of the staining was considered. We observed that 45.03% (86/191) of the EC samples showed high CHD1L expression, and its expression increased with increasing malignant degree (Table 1).

**Table 1:** Association between CHD1L expression and various clinicopathological variables of patients with EC (Chi-square test)

Variable	All cases (n=191)	CHD1L protein expression		*P* value
		Low expression (n=105)	High expression (n=86)	
Gender				0.068
Female	50	33	17	
Male	141	72	69	
Age at surgery				0.695
<60	125	70	55	
≥60	66	35	31	
Histology type				0.428
Ulcerating-type	68	33	35	
Medullary-type	74	46	28	
Fungating-type	38	20	18	
Stricture-type	11	6	5	
Clinical stages				0.003*
I	19	15	4	
II	75	47	28	
III	42	23	19	
IV	55	20	35	
Differentiation				0.590
Poor	36	18	18	
Mediate	127	70	57	
Well	27	17	10	
Tumor location				0.656
Upper	27	14	13	
Middle	140	80	60	
Lower	23	11	12	
Tumor size(cm)				0.612
<4	39	25	14	
≥4	152	80	72	
Lymph node metastasis				0.000*
None	90	27	63	
<4	35	23	12	
≥4	66	55	11	

* *P* < 0.05 was considered significant.

### Effect of CHD1L expression on clinicopathological variables in EC patients

In this study, we evaluated the associations between CHD1L expression and several clinicopathological variables in EC patients (Table 1). To confirm that CHD1L overexpression correlated with EC prognosis and clinicopathological features, the expression of CHD1L was examined by IHC in sections from 191 paraffin-embedded EC specimens. Overexpression of CHD1L in EC was significantly associated with clinical stage (*P*=0.003) and lymph node metastasis (*P*<0.01). Kaplan-Meier survival curves indicated that overexpression of CHD1L was strongly associated with poor overall survival (Figure 1C). No significant associations were observed between CHD1L expression and other clinicopathological features such as gender, age at surgery, histology type, differentiation, tumor location, or tumor size (Table 2).

**Table 2:** Clinical pathological parameters and expression of CHD1L for prognosis of 191 patients with EC by univariate survival analysis (log-rank test)

Variable	All cases	Mean survival(months)	*P* value
Gender			0.438
Female	50	40.8	
Male	141	38.9	
Age at surgery			0.690
<60	125	39.7	
≥60	66	38.8	
Histology type			0.396
Ulcerating-type	68	41	
Medullary-type	74	37.8	
Fungating-type	38	38.2	
Stricture-type	11	43.9	
Clinical stages			0.000*
I	19	45.9	
II	75	42.8	
III	42	37.2	
IV	55	30.9	
Differentiation			0.022*
Poor	36	29.9	
Mediate	127	34.4	
Well	27	44.4	
Tumor location			0.306
Upper	27	40	
Middle	140	38.35	
Lower	23	44.6	
Tumor size(cm)			0.669
<4	39	40.3	
≥4	152	39.1	
Lymph node metastasis			0.000*
None	90	45.6	
<4	35	40.3	
≥4	66	30.33	
CHD1L			0.001*
High expression	86	35.2	
Low expression	105	40.6	

* *P* < 0.05 was considered significant.

### Associations between CHD1L protein expression and the survival and prognosis of EC

Univariate survival analysis, Kaplan-Meier survival curves, and P-values for these curves were analyzed using the log-rank method. Kaplan-Meier analysis demonstrated a significant impact of well-known clinicopathological prognostic features such as clinical stage (*P*<0.01, Table 2, Figure 1D), differentiation (*P*<0.05, Table 2, Figure 1E), lymph node metastasis (*P*<0.01, Table 2, Figure 1F). A significantly shorter median overall survival was observed in patients with a high expression of CHD1L compared to patients with a low expression of CHD1L (35.2 vs. 40.6 months, *P*<0.01; Table 2, Figure 1C). A multivariate statistical analysis based on the Cox proportional hazard model was used to test the independent prognostic value of each clinicopathological feature (Table 3). Overexpression of the CHD1L protein, as well as other clinicopathological variables (clinical stage, differentiation, and lymph node metastasis) that showed significance by univariate analysis, were included in the multivariate analysis. The CHD1L protein was identified as an independent prognostic factor for poor overall survival (*P*<0.01, Table 3).

**Table 3:** Multivariate analysis on overall survival (Cox regression model)

Variable	Relative risk	95%confidence interval	*P* value
Lymph node metastasis	2.099	1.224-3.601	0.007
Differentiation	0.591	0.361-0.969	0.037
Clinical stages	1.643	1.031-2.616	0.036
CHD1L	2.817	1.533-5.180	0.031

* *P* < 0.05 was considered significant.

### CHD1L expression can be inhibited by RNA interference

**Figure 2:**
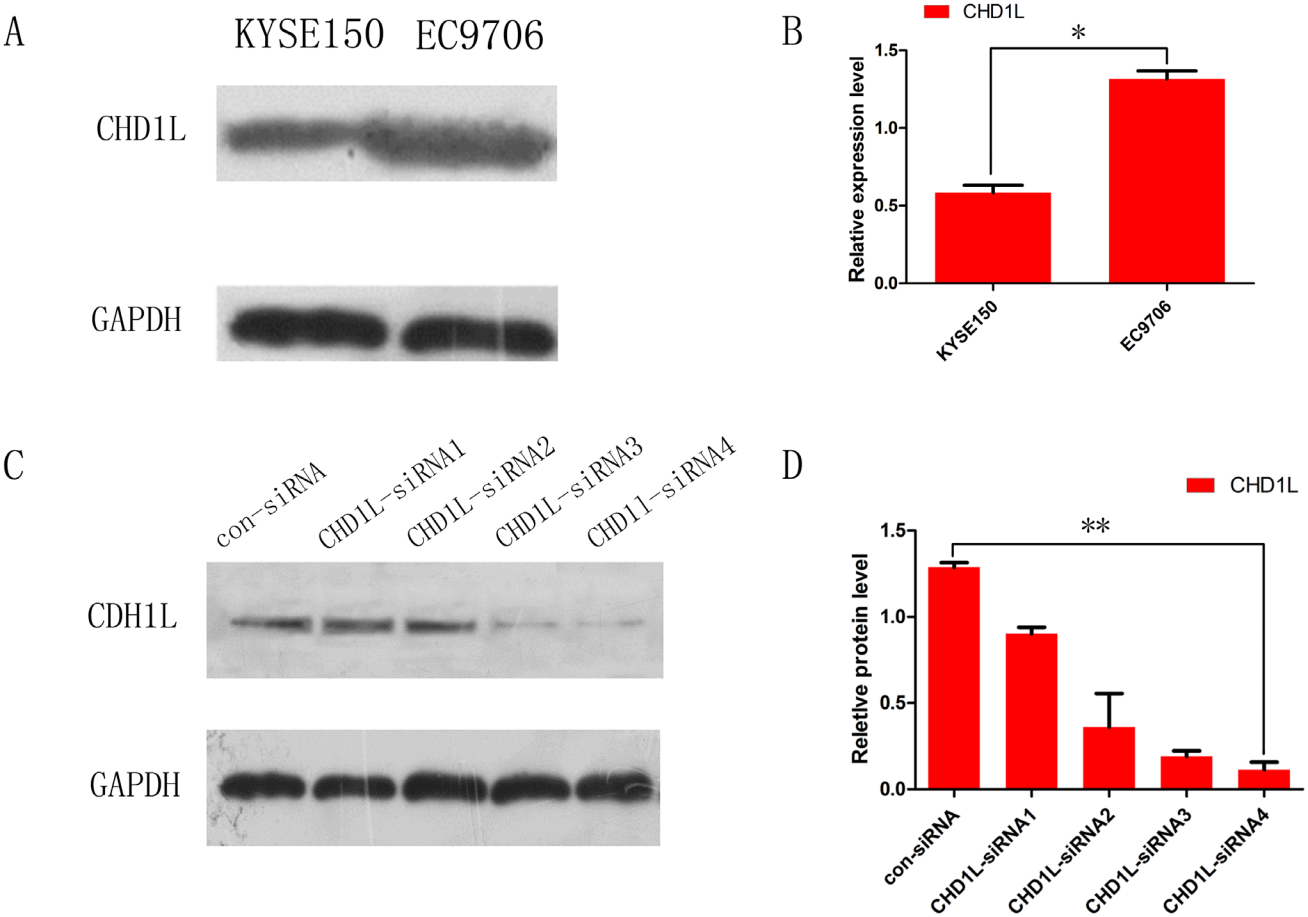
Expression of CHD1L in EC cell lines and inhibition of CHD1L expression by RNA interference **(A)** Western blot analysis of expression of CHD1L in EC cell lines (KYSE 150 and EC9706). **(B)** The bar chart demonstrates the ratio of CHD1L protein to GADPH for the above data by densitometry. The data are reported as the mean±SD of three independent experiments.(*P<0.05) **(C** and **D)** CHD1L expression was analyzed by Western blotting after EC cells were transfected with CHD1L-siRNA; CHD1L-siRNA4 achieved the most complete downregulation effect. (**P<0.01) The combination of CHD1L-siRNA4 and control-siRNA were used for all subsequent experiments.

To study the biological effects of CHD1L, we transfected CHD1L siRNA and control-siRNA into EC cells to knock down endogenous CHD1L. The EC cell line, EC9706, which expresses a much higher level of CHD1L than KYSE150 cells, was utilized in the siRNA experiment (Figure 2A&2B). The gene silencing efficiency of four siRNAs targeting CHD1L, CHD1L-siRNA1, -2, 3 and 4, was assessed by Western blot. CHD1L-siRNA4 (5’-ACAAACTCTTGCAGCCATT-3’) effectively lowered CHD1L expression by 79%, and the EC9706 cell line, CHD1L-siRNA4, and control-siRNA were used for all subsequent experiments (Figure 2C & 2D).

### CHD1L increases cell migration and cell proliferation

**Figure 3:**
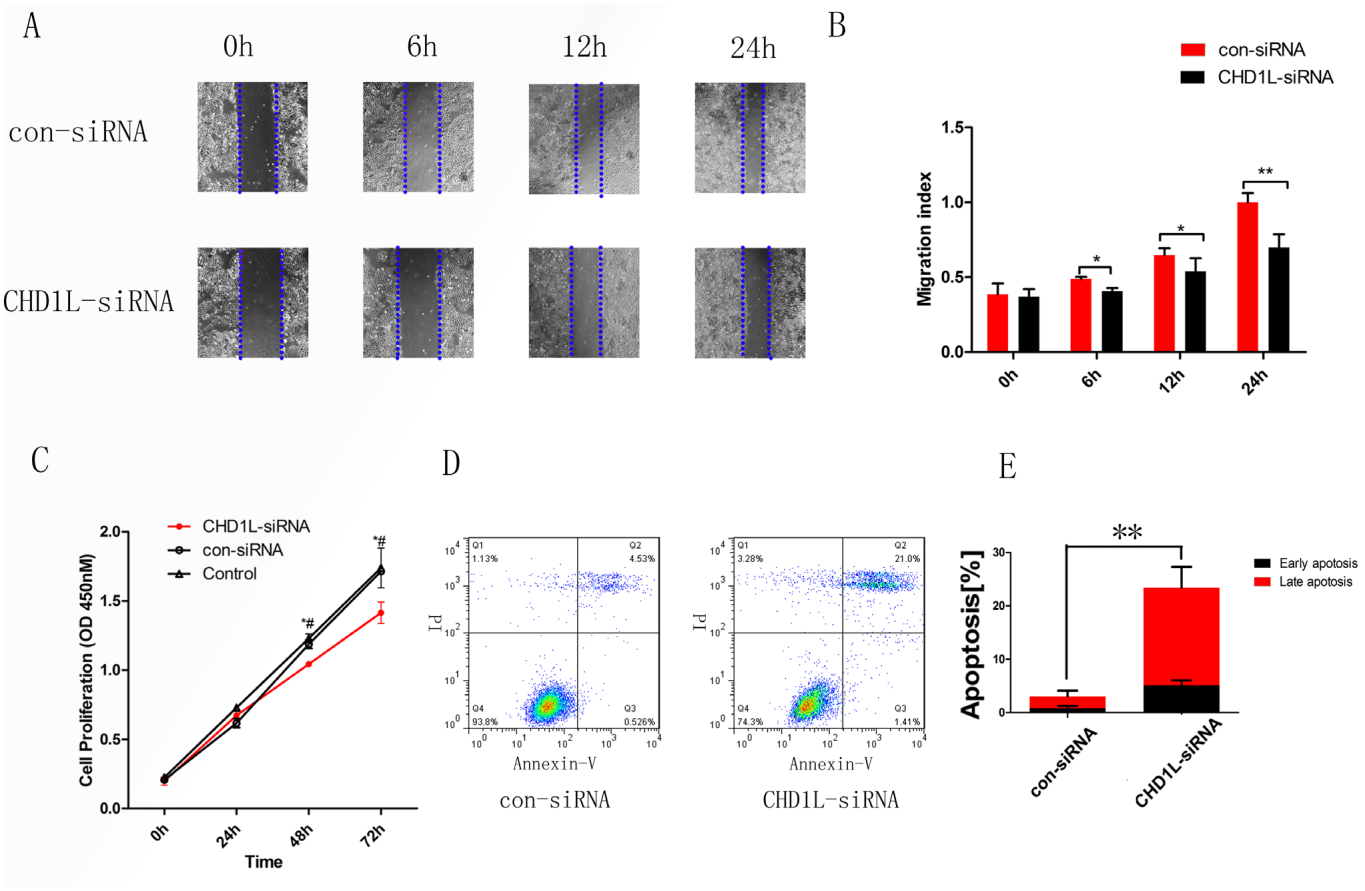
Silencing of CHD1L decreases cell migration and cell proliferation and induces cell apoptosis **(A)** Cell migration rates of CHD1L-siRNA and control-siRNA cell lines were compared using wound healing assays. Microscopic observation was demonstrated at 0, 6, 12 and 24 h after scratching the surface of cells. **(B)** The migration index bar chart demonstrates the ratio of initial width to current width. The data are reported as the mean±SD of three independent experiments. **(C)** Growth curves of EC9706 cells transfected with CHD1L-siRNA4 or control-siRNA.*: CHD1L-siRNA vs con-siRNA, *P*<0.05; #: CHD1L-siRNA vs Control, *P*<0.05 **(D)** EC9706 cells, transfected with control-siRNA and CHD1L-siRNA4, were assessed by FACScalibur according to the manufacturer’s instructions. **(E)** The bar chart represents the mean percentage of early and late apoptosis (control-siRNA vs CHD1L-siRNA4: early, 0.88% vs 5.27%; late, 2.2% vs 18.3%). (**P<0.01).

The CCK8 assay revealed that suppression of endogenous CHD1L in EC9706 cells inhibited cell viability effectively. Therefore, we supposed that knocking down the expression of CHD1L would prevent the process of migration. In the wound healing assay, the migration rate of the CHD1L-siRNA4 group was slower than that in the control group, indicating that the migration ability of the siRNA4 group was decreased by knocking down the expression of CHD1L compared to the control group. (Figure 3A, 3B & 3C).

### Inhibition of CHD1L induces apoptosis in EC cells

Decreased apoptosis is another major mechanism of oncogenes involved in cancer development; we hypothesized that CHD1L might be involved in apoptosis in EC, and we assessed the effect of CHD1L on survival of EC cells by flow cytometry. Based on our results, we concluded that compared with control-siRNA-transfected cells, there were more apoptotic cells (both early and late apoptosis) in the CHD1L-siRNA4 group. These results indicate that CHD1L inhibits the progression of apoptosis in EC cells (Figure 3D & 3E).

## DISCUSSION

Despite improved diagnostic and therapeutic strategies, EC is one of the deadliest human malignancies. At present, the prognosis of EC remains unsatisfactory, and EC represents an invasive, rapidly proliferating tumor; therefore, it is necessary to identify prognostic biomarkers [18–21] that are independently correlated with tumor prognosis and aggressiveness [22, 23]. Recent reports demonstrate that CHD1L is frequently amplified in HCC. CHD1L belongs to the SNF2 superfamily, containing an SNF2-N domain and a helicase superfamily domain [24, 25]. Therefore, CHD1L has been hypothesized to perform important roles in transcriptional regulation, maintenance of chromosome integrity, and DNA repair. In addition, amplification of CHD1L has been observed in various solid tumors, including breast cancer, colorectal cancer, bladder cancer, nasopharyngeal cancer, and HCC. In this present study, we aimed to analyze CHD1L protein expression in tumor tissues and evaluate its prognostic significance for EC. The results of this study demonstrated that CHD1L was overexpressed in EC tissues, opposed to normal tissues. However, to date, the expression and clinical significance of CHD1L in EC had not been explored. We found a significant correlation between CHD1L expression and poor clinical outcome, independent of other characteristics. In addition, our results indicate that CHD1L expression is a potential novel prognostic marker for EC [26–28]. These results correspond to those of previous studies in other types of human cancers. To investigate if CHD1L expression might be involved in the progression of EC, CHD1L expression levels and clinicopathological characteristics of 191 patients with EC were investigated through immunohistochemistry. We found that high CHD1L expression was substantially correlated with lymph node metastasis and clinical stage. Furthermore, we demonstrated the prognostic significance of CHD1L protein in EC, demonstrating that CHD1L protein expression was inversely related to overall survival. The Kaplan-Meier analysis revealed that CHD1L overexpression, in addition to clinical stages and differentiation, predicted poor survival. Patients with higher expression of CHD1L had shorter survival. Multivariate analysis using the Cox proportional hazards model indicated that CHD1L was an independent prognostic factor for survival in EC patients. Functional studies suggest that the oncogenic function of CHDL1 in HCC tumorigenesis is served through unleashed cell proliferation, migration, and inhibition of apoptosis [29]. The proliferative role of CHD1L in EC can be efficiently inhibited by CHD1L siRNA. These results concur with previous findings in HCC cell lines. In our study, CHD1L played an inhibitory role in apoptosis, and the oncogenic function of CHD1L was associated with its antiapoptotic ability [30]. The inhibition of apoptosis is one of the major mechanisms in cancer development and ultimately extends cell survival, allowing for the accumulation of genetic instability and mutations [31]. Although radical surgery is an curative option for EC patients, the recurrence rate is high, owing to the possibility of metastasis. Furthermore, we discovered that overexpression of CHD1L in cell lines correlated with a tendency of lymph node metastasis by showing decreased migration potential in cells where CHD1L was knocked down. In conclusion, our data suggest that CHD1L plays a significant role in EC pathogenesis by the promotion of cell proliferation and migration and the inhibition of apoptosis. However, this was a retrospective cohort study and we did not perform a metastasis assay *in vivo* model; further studies will be needed in order to thoroughly understand the molecular mechanisms of CHD1L involved in EC progression and prognosis. Thus, further research may be focused on the molecular mechanism and the development of novel approaches targeting CHD1L for effective tumor management.

## PATIENTS AND METHODS

### Patients and cell lines

Paraffin-embedded tissue samples from 191 EC patients were received from the Department of Thoracic Surgery, Fujian Provincial Tumor Hospital, Fujian Province, China, between January 1, 2004 and October 1, 2006. The median and mean age of the population are 56 (34, 80) and 56±9.57. The sex ratio is 3/8 (female/male). Primary EC specimens were obtained with informed consent from patients who had undergone esophageal surgery for EC at the Department of Surgery, Fujian Provincial Tumor Hospital, (Fujian Medical University, Fujian, China). All of the EC samples (n=191) histological type is esophageal squamous cell carcinoma, which is also the most common histological type of esophageal cancer in China. All biopsies were histologically confirmed by two pathologists in a blind manner. All specimens were handled and made anonymous according to accepted ethical and legal standards. None of the patients were treated with preoperative immunotherapy, radiation, or chemotherapy. Clinical stage was determined according to the pathological tumor-node-metastasis (pTNM) system (7th edition, AJCC/UICC 2009), and the histological type was selected according to the World Health Organization (WHO) criteria.

Human EC cell line EC9706 was a generous gift from the Tianjin Lung Cancer Institute, Tianjin Medical University General Hospital. EC9706 cells were originally purchased from ATCC (Virginia, USA). The other human esophageal squamous-cell carcinoma cell line.

KYSE150 was a gift from Dr. Yutaka Shimada, who established this cell line at the Department of Surgery and Surgical Basic Science, Graduate School of Medicine, Kyoto University, Japan. The two esophageal cancer cell lines, KYSE150 and EC9706, were cultured in RPMI 1640 complete growth medium under the recommended conditions.

### Immunohistochemistry (IHC)

Immunohistochemistry (IHC) studies were carried out using the standard streptavidin-biotin-peroxidase complex method. For antigen retrieval, microwave pretreatment was done with a 0.01 mol/L EDTA buffer (pH 9.0) for 30 min. For antigen retrieval, microwave pretreatment was done with a 0.01 mol/L EDTA buffer (pH 9.0) for 30 min. The sections were then incubated with rabbit monoclonal anti-CHD1L antibody (Abcam, Cambridge, UK) in a dilution of 1:150 at 4°C overnight in moist chambers. The slides were sequentially incubated with biocatalytic goat anti-mouse IgG (1:100 dilution; ZSGB Biotechnology) and streptavidin-peroxidase conjugate for 30 minutes each at room temperature. Isotope-matched human IgG was utilized in each case as a negative control. Finally, the 3, 5-diaminobenzidine (DAB) substrate kit (ZSGB) was used for color development, followed by Mayer's hematoxylin counterstain.

For evaluation of CHD1L staining, a semiquantitative scoring criterion was utilized, in which both staining intensity and positive cell percentage were contained. A staining index (with values from 0 to 12) was created for the intensity of CHD1L staining as follows: (0=negative, 1=weakly positive, 2=positive, 3=strongly positive) times the proportion of immunopositive tumor cells (0%=0; <10%=1; ≤10% to <50%=2; ≤50% to <75%=3; ≥75%=4). We evaluated the expression level of CHD1L by staining index (scored as 0, 1, 2, 3, 4, 6, 9 or 12) using this method. The staining index score was graded as negative (scored as 0–3) or positive (4–12) expression. A minimum of 300 epithelial cells was counted for each case. All histological evaluations were conducted in a double-blind manner by two expert pathologists.

### Western blot analysis

Cell proteins were promptly homogenized in a homogenization buffer containing 1 M TrisHCl pH 7.5, 1% Triton X-100, 1% Nonidet P-40 (NP-40), 10% sodium dodecyl sulfate (SDS), 0.5% sodium deoxycholate, 0.5 M EDTA, and 1 mM PMSF, and centrifuged at 10, 000×g for 30 min to collect the supernatant. Protein concentrations were established using a BSA protein assay (Beyotime, SH, China). The total cellular protein extracts were separated by sodium dodecyl sulfate–polyacrylamide gel electrophoresis (SDS-PAGE) and transferred to polyvinylidene difluoride filter (PVDF) membranes (Roche, Basel, Switzerland). After the membranes were blocked in 5% nonfat milk in TBST (150 mM NaCl, 20 mM Tris, 0.05% Tween 20) for 2 h, they were incubated with their primary antibodies overnight at 4°C. The membranes were washed with TBST three times for 5 min each, and the horseradish-peroxidase-linked IgG was used as the secondary antibody for 2 h at room temperature. The membrane was developed using the ECL detection systems. The experiments were performed on three separate occasions. The densitometry data were measured and analyzed using ImageJ software.

### Small interfering RNA transfection

EC cells were transfected with double-stranded small interfering RNA (siRNA) (Ribobio, GZ, China) with Lipofectamine 3000 Reagent (Thermofisher), according to the manufacturer’s instructions. The CHD1L-specific siRNA target sequences were as follows: CHD1L-siRNA#1, 5’-GTATTGGACATGCCACGAAA-3’; CHD1L-siRNA#2, 5’-TATTGGACATGCCACGAAA-3’; CHD1L-siRNA#3, 5’- CAAGAGAAGGAGACTCATA-3’; and CHD1L-siRNA#4, 5’-ACAAACTCTTGCAGCCATT-3’. At 48 h after transfection, the gene silencing effect was measured using Western blot. Three independent experiments were carried out.

### Apoptosis analysis

Apoptosis analysis used the TACS·XL DAB *in Situ* Apoptosis Detection Kit (Trevigen) according to the manufacturer’s instructions. Using the Annexin V Cell Apoptosis Analysis Kit (Sungene Biotech), apoptosis of EC9706 cells were examined using FACScalibur (BD Biosciences, USA), and the acquired data were analyzed using Flow Jo 7.6 software (Tree Star, Inc, USA).

### Wound healing assays

Cell migration was evaluated by measuring the movement of cells into a scraped, acellular area created by a 200-mL pipette tube. The extent of wound closure was observed after 6, 12, and 24 h and photographed under a microscope.

### Cell proliferation assays

Cell growth was monitored using the commercial Cell Counting Kit (CCK) -8 (Dojindo, Kumamoto, Japan) in accordance with the manufacturer’s instructions. Briefly, cells were plated onto a 96-well plate at a density of 5×10^3^ cells/well and grown overnight. Assessment of cell proliferation was done at initiation, 24, 48, and 72 h. The CCK-8 reagent (10 μL) in medium (1:10) was added to each well and allowed to react at 37°C for 1.5 h. OD was set at 450 nm. The average of five parallel wells was used for each group. The experiment was performed three times.

### Statistical analysis

Statistical analysis was performed using SPSS software (SPSS Standard Version 19.0, SPSS Inc. Chicago, IL). The association of CHD1L protein expression with clinicopathological features and the correlation with CHD1L gene amplification in EC patients were assessed using the Chi-square test. Survival curves were evaluated by the Kaplan-Meier method and compared using the log-rank test and Gehan-Breslow-Wilcoxon test. A Cox regression analysis was performed to assess the significance of variables for survival. Two-sided P-values <0.05 were regarded as statistically significant. Graphs were drawn using GraphPad Prism Version 5.0 (GraphPad Software Inc., San Diego CA).
